# Cognitive and Emotional Empathy in Individuals with Spinal Cord Injury

**DOI:** 10.1155/2019/1312934

**Published:** 2019-02-10

**Authors:** Veronica Guadagni, Marco Sarà, Massimiliano Conson, Antonio Carolei, Simona Sacco, Silvia Vadini, Caterina Pistarini, Arcangelo Barbonetti, Giuseppe Iaria, Francesca Pistoia

**Affiliations:** ^1^Department of Physiology and Pharmacology Cumming School of Medicine, University of Calgary, Calgary, Alberta, Canada; ^2^Hotchkiss Brain Institute, Cumming School of Medicine, University of Calgary, Calgary, AB, Canada; ^3^O'Brien Institute for Public Health, Cumming School of Medicine, University of Calgary, Calgary, AB, Canada; ^4^Department of Psychology and Alberta Children's Hospital Research Institute, University of Calgary, Calgary, Alberta, Canada; ^5^IRCCS “San Raffaele Pisana”, Via di Valcannuta, 247, I-00166 Rome, Italy; ^6^Neuropsychology Laboratory, Department of Psychology, University of Campania Luigi Vanvitelli, Caserta, Italy; ^7^Neurological Institute, Department of Biotechnological and Applied Clinical Sciences, University of L'Aquila, Italy; ^8^IRCCS ICS Maugeri, Genova Nervi, Italy; ^9^Spinal Unit, San Raffaele Sulmona Institute, 67039 Sulmona, Italy

## Abstract

**Background:**

Empathy has been conceptualized as comprising a cognitive and an emotional component, the latter being further divided into direct and indirect aspects, which refer, respectively, to the explicit evaluation of the observer's feelings while attending someone in an emotional situation and to the physiological response of the observer. Empathy has been previously investigated in several neurological disorders.

**Objective:**

This study is aimed at investigating empathy in patients with spinal cord injury (SCI). We hypothesize that, due to deafferentation following their injury, SCI patients will display difficulty in the processing of emotional stimuli and blunted empathic responses as compared to healthy controls.

**Materials and Methods:**

20 patients with spinal cord injury (SCI) (12 males and 8 females, mean age = 50.9, standard deviation (SD) = 16.1 years; mean education = 10.9, SD = 4.1 years) were included in the study and compared to 20 matched healthy subjects. Participants were investigated using the State-Trait Anxiety Inventory (Form Y) (STAI-Y), the Beck Depression Scale, and the Toronto Alexithymia Scale. Moreover, participants were further evaluated by means of the Interpersonal Reactivity Index (IRI), which explores both cognitive and emotional aspects of empathy, and through an experimental protocol based on the use of a modified version of the computerized Multifaceted Empathy Test (MET) to evaluate emotional (direct and indirect) empathy and the ability to judge the valence of complex emotional scenes.

**Results:**

As compared to healthy controls, SCI patients reported higher scores on the *Perspective-Taking* subscale of the IRI, while, on the modified MET, they were less accurate in identifying the valence of neutral scenes, notwithstanding their spared direct and indirect emotional empathy ability. Furthermore, we found a significant negative correlation between the time interval since injury and the direct emotional empathy scores on the positive images, as well as a negative correlation with the indirect emotional empathy scores on both positive and neutral images, indicating a blunting of the empathic responses as time elapses.

**Conclusion:**

Results suggest that SCI patients, when analyzing the meaning of emotional stimuli, tend to rely on a cognitive empathy strategy rather than on emotion simulation.

## 1. Introduction

Empathy is an evolutionary high-order cognitive ability that developed in primates, so they could understand the state of mind and thoughts of others with the aim of predicting future behaviour [1]. After an extensive debate about the nature of this construct, researchers have reached agreement on the dual nature of empathy and have conceptualized it as comprising a cognitive and an emotional component [2–4]. Cognitive empathy (also referred to as the theory of mind) refers to an individual's ability to understand another person's perspective, feelings, and state of mind [5]. Emotional empathy refers to the ability to understand the emotions of others by vicariously sharing them [1]. Emotional empathy can be manifested as increased feelings of distress while observing someone else in a negative situation and does not require an explicit understanding of why the individual is suffering. The most primitive precursor of emotional empathy is *emotional contagion*, which can be experienced even by one-day-old babies, who do not have a clearly developed ability to recognize the difference between self and other people [6]. Cognitive empathy, on the other hand, develops later, in the preschool years [7], and is a fundamental component of prosocial behaviour.

Davis [8] demonstrated insights into the multidimensional aspect of empathy. Based on his theory, Davis developed a questionnaire—the Interpersonal Reactivity Index (IRI)—which assessed four different aspects of empathy: (1) *Perspective-Taking*, which is the ability of an individual to put himself in someone else's shoes; (2) *Fantasy*, which is the ability of an individual to identify with fictional characters; (3) *Empathic Concern*, defined as the feelings of concern and compassion while attending others in emotional situations; and (4) *Personal Distress*, which refers to the negative feelings of anxiety and distress that the observer experiences while others are suffering. Within the recent categorization of empathy, Davis' dimensions of *Perspective-Taking* and *Fantasy* fit in the cognitive empathy component since they describe the ability of individuals to adopt different perspectives, or points of view [8], while *Empathic Concern* and *Emotional Distress* fit in the emotional empathy component since they describe individuals' responses to the observed emotional experiences of others [8, 9]. The IRI is still used as the traditional questionnaire to assess empathy, with higher scores on the subscales indicating greater empathic abilities.

In more recent years, Dziobek and colleagues [2] further divided emotional empathy into direct and indirect components. The direct emotional empathy component refers to the explicit evaluation of the observer's feelings while attending someone else in an emotional situation (e.g., how much empathy/concern do you feel for the person in distress?). The indirect emotional empathy, on the other hand, is the physiological activation of the observer (e.g., my heart is beating faster when I see this person in distress). The same authors developed a computerized empathy task named the Multifaceted Empathy Test (MET) [2], measuring two such aspects of emotional empathy. The original MET [2] was first used to test empathy in adults with Asperger syndrome, thereafter being used to investigate empathy in other clinical populations showing complex patterns of cognitive and emotional empathy impairment, including frontotemporal dementia [10], borderline personality disorder [11], autism [12], and major depression [13]. The MET was also used to test healthy controls [14].

Previous studies have shown impairments in emotional processing in individuals suffering from spinal cord injuries [15–17]. Spinal cord injuries (SCI) are defined as acute (traumatic) or chronic (due to comorbid diseases) damage to the spinal cord [18]. The traumatic etiology is the most prevalent (39 cases per one million individuals in North America) [19], with the highest incidence in males between 15 and 29 years of age (79.8%), even though the prevalence of injuries is rapidly increasing in the population above 60 years of age due to falls and increasing lifespan [18, 20]. The initial local trauma is due to spinal cord compression and damage of the small vasculature, often followed by hemorrhages and a cascade of events that lead to neuronal death [18]. Further, the formation of scar tissue and other molecular mechanisms often impede axonal growth and motor recovery [21]. According to the American Spinal Injury Association (ASIA) impairment scale [22], there are four levels of injury severity in SCI, based on the level of both motor and sensory impairments shown by the patient. Grade A (complete) on the scale is the highest level of impairment, characterized by a complete lack of motor and sensory function below the level of injury (including the anal area), with <5% chance of walking at one year postinjury [23]. Grade B (sensory incomplete) is the second highest level, with sensory but not motor function being preserved below the level of the injury, with some sensation (including anal sensation) being preserved. In grade C (motor incomplete), motor function is preserved at the caudal sacral segments for voluntary anal contraction, and around 50% of the muscles below the level of injury have some spared function although they are not strong enough to move against gravity. In grade D (motor incomplete), more than 50% of the muscles that are spared below the level of injury are strong enough to move against gravity. Finally, all neurological functions are restored in grade E (normal). Higher ASIA grades are associated with greater loss of independence and poorer quality of life for both the patients and the patients' caregivers [24], while individuals classified with grades below B have the greatest chance of walking at discharge [23]. In addition to the loss of sensorimotor function, patients with lesions in the thoracic area can also develop autonomic dysreflexia, a life-threatening condition characterized by aberrant responses of the sympathetic system, with high blood pressure, altered heart rate, and feelings of anxiety and distress. Previous studies have reported an association between sensorimotor deafferentation and sympathetic deregulation in SCI patients and the presence of emotional deregulation and mood disorders, leading to poor quality of life and impaired social behaviour [15, 25, 26].

In this study, moving from the multidimensional view of the construct of empathy [2, 8], we tested the hypothesis that emotional processing, and particularly high-order emotional processes such as empathy, is impaired in patients with SCI. Previous studies [16] have found impairments in emotional processes in SCI patients. When asked to judge emotionally evocative scenes, individuals with SCI, as compared to matched healthy controls, were found to have difficulty in judging their own emotional response to complex scenes eliciting fear and anger. These findings endorsed the classical theories of emotions, and particularly the physiological ones, such as the James-Lange theory of emotion, which suggest that the perception we have of emotions is influenced by our physiological responses to external stimuli, with the physiological responses preceding and therefore influencing the generation of emotions [27]. In SCI patients with sensory deafferentation and autonomic dysreflexia, the impaired ability to perceive their own internal state (interoception) may contribute to the inability to label emotions according to first-hand experiences correctly. In the same vein, Nicotra et al. [15], using functional magnetic resonance imaging (fMRI), confirmed the existence of significant differences between SCI patients and controls when evaluating emotional pictures and particularly pictures depicting threat. The authors found differences in blood-oxygen-level-dependent (BOLD) signal change in brain areas such as the posterior cingulate cortex, the motor cingulate cortex, the subgenual cingulate cortex, and the adjacent ventromedial prefrontal cortex, areas involved in the processing of emotional stimuli. Altogether, these studies support the hypothesis that deafferentation, which characterizes SCI, leads to impairments in the processing of emotional stimuli.

To our knowledge, this is the first study testing empathy in SCI patients. Here, we combined the use of a self-report measure, the Davis IRI [8], and a modified version [14] of the computerized [2] task of the MET to evaluate cognitive empathy and emotional (direct and indirect) empathy together with the ability to judge the valence of complex emotional scenes. We hypothesize that, due to deafferentation, SCI patients, as compared to healthy controls, will show difficulty in evaluation of the valence of emotional scenes together with blunted empathic responses.

## 2. Materials and Methods

### 2.1. Participants

Twenty patients with SCI (12 males and 8 females, mean age = 50.9, SD = 16.1 years, mean education = 10.9, SD = 4.1 years) were recruited through the IRCCS Foundation Hospital Salvatore Maugeri, Pavia, Italy, and the Spinal Unit, San Raffaele Sulmona Institute, Italy, and matched by age, sex, and education with 20 healthy controls (12 males and 8 females, mean age = 50.0, SD = 15.9 years, mean education = 10.4, SD = 4.1 years). At the time of testing, none of the controls were taking psychoactive medication nor had a history of any diagnosed cardiovascular or psychiatric disorder as assessed by a self-report questionnaire. The neurological assessment carried out on all the patients excluded the presence of associated cognitive deficits. The cause of the SCI damage was traumatic in most of the cases (*n* = 16), followed by vascular (*n* = 3) and neurodegenerative damage (*n* = 1) in the remainder. Most of the patients were graded as B (*n* = 10) on the American Spinal Injury Association (ASIA) impairment scale [22], while the others were graded as A (*n* = 7) or C (*n* = 3). The study was carried out in conformity with the Declaration of Helsinki (statement of ethical principles regarding human experimentation), with no discomfort being experienced by the patients and control subjects.

### 2.2. Procedures

#### 2.2.1. Questionnaires

All participants were assessed by means of the following assessment tools: the State-Trait Anxiety Inventory (STAI) [28] in both STAI Y-1 (State) and STAI Y-2 (Trait) forms, to investigate symptoms of anxiety (scores > 48 are indicative of problematic anxiety); the Beck Depression Scale (BDI) [29] to check for the presence of depressive symptoms (scores > 17 are indicative of moderate depression); and the Toronto Alexithymia Scale (TAS 20) [30] to ensure participants were not affected by alexithymia (scores > 60), a condition characterized by difficulties in identifying, processing, or describing an individual's own emotions, subjective feelings, and bodily sensations, as well as emotions expressed by others during social interaction [31, 32]. These questionnaires were used to exclude psychiatric comorbidities, such as depression [33], anxiety [34], and alexithymia [31], all conditions in which emotional processing may be impaired.

In addition, participants were assessed through the IRI [8] as a traditional measure of empathy. The IRI does not have a clinical cut-off. Higher scores on the subcomponents (*Perspective-Taking*, *Fantasy*, *Empathic Concern*, and *Personal Distress*) indicate greater empathic abilities. SCI patients' and controls' means and standard deviations (SD) are reported in Table 1.

#### 2.2.2. Experimental Tasks

The experimental task consisted of a modified version of the MET [2] that was previously used in other studies on different populations [14]. We selected 120 coloured images from the International Affective Picture System (IAPS) [35], of which 40 people were depicted in positive scenes (valence *M* = 8.0; arousal *M* = 5.1), 40 in neutral scenes (valence *M* = 5.0; arousal *M* = 3.6), and 40 in negative (valence *M* = 2.0; arousal *M* = 6.0) scenes, as categorized by the IAPS normative data. We then created two parallel versions of the same task that were used in a counterbalanced order in the testing sessions for both patients and controls. The two tasks each consisted of 60 images, matched for valence and arousal as provided by the IAPS normative data.

**Figure 1:**
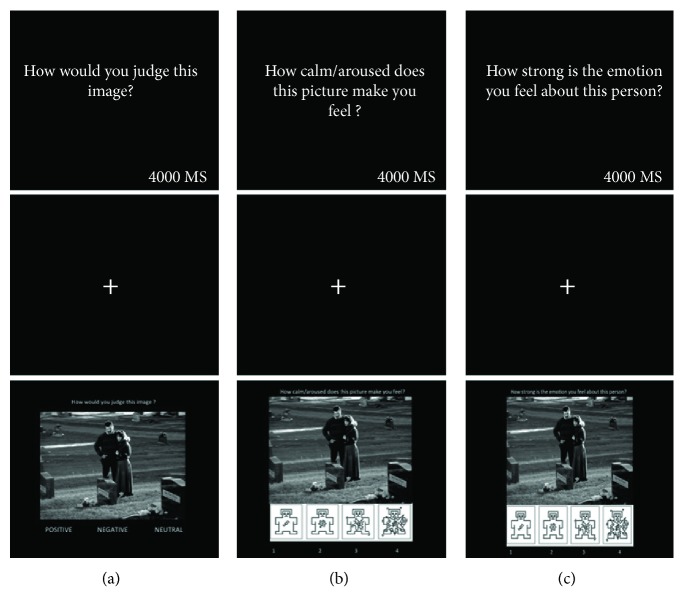
The figure depicts a sample of the computerized emotional empathy task assessing the ability of participants to recognize the image valence (a), emotional empathy indirect (b), and emotional empathy direct (c).

In each task, the images were presented three times, each time paired with a different question, measuring (i) the ability to judge the valence of the scene as positive, negative, or neutral (i.e., “How would you judge this image?”); (ii) direct emotional empathy (i.e., “How strong is the emotion you feel about this person?”); and (iii) indirect emotional empathy (i.e., “How calm/aroused does this picture make you feel?”). Thus, each task consisted of 180 trials. In each trial, an image was shown, and participants were required to give their responses as quickly as possible, using the keyboard. Participants responded on a Likert scale from 1 to 4 and were reminded how to respond with a reduced version of the Self-Assessment Manikin (SAM) valence scale positioned at the bottom of each image [36]. Participants were required to answer the three questions on randomized blocks of ten trials each. Each task included 18 blocks, and each block had only one question posed. Each block was initiated with an on-screen query for the duration of 4000 milliseconds, followed by a fixation cross and the image stimulus which remained on-screen until participants responded, or until ten seconds had elapsed. In each experimental task, the presentation of the blocks was randomized, and the task started with a practice session consisting of six trials. Responses for the SCI patients were recorded by an expert examiner, by pressing the corresponding keys on the computer keyboard. The examiner was unaware of the purpose and predictions of the experiment.  Figure 1 contains samples of trials used in the tasks.

#### 2.2.3. Data Analysis

Questionnaire scores for SCI patients and controls, as well as sex differences in age, education, anxiety, depression, and IRI scores in both SCI patients and controls, were compared with independent sample *t*-tests. We computed participant accuracy in evaluating the valence of a scene as the sum of the instances that participants correctly responded “positive” to a positive image, “neutral” to a neutral image, and “negative” to a negative image. We used independent sample *t*-tests to compare differences between the two groups (SCI patients and controls), in (i) overall accuracy and (ii) accuracy for the positive, neutral, and negative images separately. Moreover, to verify whether the two groups tended to misattribute the valence of the scenes towards a specific response category (valence attribution error bias), we compared valence attribution errors (positive scenes judged as neutral or negative, neutral scenes judged as negative or positive, and negative scenes judged as neutral or positive) in the two groups separately by performing paired sample *t*-tests.

We then computed the average direct and indirect emotional empathy responses to positive, neutral, and negative images and compared them between the two groups using the Mann-Whitney *U* test. We used a Spearman correlation analysis to further explore the association between emotional empathy scores and questionnaire scores in the SCI patients and control groups separately and a Pearson correlation analysis to explore the association between SCI severity (ASIA grade) and emotional empathy scores and questionnaire scores exclusively in the SCI patients. To further control for empathic responses as related to correct attribution of valence, we computed average direct and indirect emotional empathy scores for images that were correctly and incorrectly judged according to their valence and compared them between the two groups using the Mann-Whitney *U* test. To further analyze the association between accuracy and scores on the IRI, we clustered together the *Perspective-Taking* and *Fantasy* subscales into a “cognitive empathy IRI variable” and clustered the *Empathic Concern* and *Personal Distress* subscales into an “emotional IRI variable.” We then used a Pearson correlation analysis to examine the association between accuracy in evaluating the valence of a scene and scores obtained on the questionnaires. All analyses were two-tailed, with statistical significance set at *p* < 0.05.

## 3. Results

The comparisons between SCI patients and controls in the responses to the questionnaires are reported in Table 1. The only statistically significant difference was found in the *Perspective-Taking* scale of the IRI, with SCI patients (*M* (SD) = 24.6 (3.7)) reporting higher scores as compared to controls (*M* (SD) = 21.2 (4.0)), *t*_38_ = 2.751, *p* = 0.009. Furthermore, we used independent sample *t*-tests to assess sex differences in age, education, anxiety, depression, alexithymia, and IRI scores in both SCI patients and controls, and no statistically significant differences were found (all *p* > 0.05).

The independent sample *t*-tests on overall accuracy between the groups revealed no statistically significant differences between SCI patients and controls (*p* > 0.05); however, when separating the accuracy responses to the three different valences (positive, neutral, and negative), SCI patients (*M* (SD) = 8.2 (2.9)) had lower correct responses when evaluating neutral stimuli as compared to the controls (*M* (SD) = 11.0 (3.2), *t*_38_ = 2.888, *p* = 0.006). No statistically significant differences were found when comparing the groups on accuracy responses to positive or negative stimuli (*p* > 0.05). Moreover, when comparing valence attribution errors in the two groups, both patients and controls tended to incorrectly judge positive scenes as neutral rather than negative (SCI: *t*_19_ = −2.285, *p* = 0.034; CON: *t*_19_ = −4.849, *p* = 0.0001) and neutral scenes as positive rather than negative (SCI: *t*_19_ = 3.030, *p* = 0.007; CON: *t*_19_ = 4.466, *p* = 0.0001), whereas negative scenes could be judged as neutral or positive without significant differences (all *p* > 0.05).

The Mann-Whitney *U* test on the direct emotional empathy scores did not reveal significant differences between the groups in response to positive, neutral, and negative images (all *p* > 0.05). Similarly, the Mann-Whitney *U* test on the indirect emotional empathy scores did not show significant differences between the groups in response to positive, neutral, and negative images (all *p* > 0.05). The Spearman correlation analysis revealed no statistically significant correlation between the indirect and direct empathy scores and questionnaires for both SCI patients and controls (all *p* > 0.05) and no statistically significant association between the degree of SCI severity as measured by the ASIA impairment scale and emotional empathy scores (both direct and indirect) or questionnaire scores in SCI patients (all *p* > 0.05).

The Mann-Whitney *U* test on the direct emotional empathy scores, of both correctly and incorrectly judged images, did not reveal significant differences between the two groups in response to positive, neutral, and negative images (all *p* > 0.05). Similarly, the Mann-Whitney *U* test on the indirect emotional empathy scores, of both correctly and incorrectly judged images, did not reveal significant differences between the two groups in response to positive, neutral, and negative images (all *p* > 0.05).

The Pearson correlation analysis revealed that overall accuracy in evaluating the valence of a scene was negatively correlated with the BDI (*r* = −0.451, *p* = 0.046), IRI (*r* = −0.527, *p* = 0.017), and *Fantasy* scale of the IRI (*r* = −0.604, *p* = 0.004) exclusively in SCI patients. No significant correlations were found in the controls (all *p* > 0.05). Moreover, we clustered together the *Perspective-Taking* and *Fantasy* scales into a “cognitive empathy IRI variable” and clustered the *Empathic Concern* and *Personal Distress* into an “emotional IRI variable.” The newly computed “cognitive IRI variable” was significantly correlated to overall accuracy (*r* = −0.537, *p* = 0.015) and to the “emotional IRI variable” (*r* = 0.448, *p* = 0.047) in SCI patients. Conversely, the “cognitive IRI variable” was only correlated to the “emotional IRI variable” in the controls (*r* = 0.660, *p* = 0.002).

### 3.1. Supplementary Analyses: Time from Lesion in SCI Patients

In order to test whether patient performance on direct and indirect emotional empathy tasks could be influenced by the time interval between the event causing SCI and the time of the present evaluation, a Spearman correlation analysis was conducted between time elapsed since SCI lesion (in months) and performance, on the two empathy tasks—separately for positive, negative, and neutral images. The results revealed a significant negative correlation between months elapsed since SCI lesion and direct emotional empathy scores on the positive images (rho = −0.666, *p* = 0.002) and indirect emotional empathy scores on both positive images (rho = −0.665, *p* = 0.002) and neutral images (rho = −0.523, *p* = 0.022); no other correlation was statistically significant (all *p* > 0.05). However, the correlation between months from lesion and indirect emotional empathy scores on neutral images did not survive Bonferroni correction for multiple comparisons (*α* = 0.05/5 = 0.01).

## 4. Discussion

In the current study, we investigated the processing of empathy in SCI patients as compared to healthy controls following the conceptualization of empathy as comprised of a cognitive and an emotional component [3, 8]. To meet this aim, we employed both the Davis IRI [8] (the most common psychometric tool to assess individuals' empathy) and a recently validated computerized experimental task measuring emotional empathy, the MET [2, 14]. Our findings showed that SCI patients reported higher scores than the healthy controls on one of the two cognitive empathy subscales of the IRI, the *Perspective-Taking* scale. On the behavioural experiment, SCI patients proved to be less accurate than the controls in identifying the valence of a scene, but only when neutral scenes were presented and without any valence attribution error bias. However, notwithstanding reduced accuracy in the identification of the valence of emotional scenes, no differences were found between SCI patients and controls in the average scores obtained in the two behavioural tasks assessing direct and indirect emotional empathy. Further analysis on correctly and incorrectly identified scenes confirmed that SCI patients and controls were not different in their empathic responses regardless of their ability to judge correctly the valence of a scene. Interestingly, the overall accuracy in evaluating the valence of a scene in SCI patients was negatively correlated with the *Fantasy* scale of the IRI, with the “cognitive IRI variable” that was computed (clustering of the *Perspective-Taking* and *Fantasy* scales), and with the scores obtained in the depression scale (BDI).

Altogether, these findings indicate that SCI patients experience some difficulty in evaluating the valence of an emotional scene, particularly when processing neutral scenes, as compared to healthy controls. However, this impairment does not seem to affect their ability to process direct and indirect emotional empathy. The latter finding was unexpected and rather interesting, because it demonstrates a dissociation between patients' ability to attribute valence to an emotional scene and their ability to process higher-order emotional processes such as empathy. Furthermore, SCI patients reported higher scores on the *Perspective-Taking* scale as compared to the controls, and their valence attribution accuracy was negatively correlated with their self-reported ability to take the perspective of fictional characters (*Fantasy*) and more generally with the cognitive empathy component of the IRI.

This dissociation between defective valence identification of emotional scenes and spared emotional empathy is in line with previous data demonstrating how SCI patients experience difficulty when processing emotional stimuli [15, 16], due to their often high level of injury in the spinal cord that generates a deafferentation of the central nervous system (CNS) from the periphery. In particular, our results of impaired valence attribution to ambiguous (neutral) emotional scenes are consistent with the findings of Pistoia et al. [16] who tested the hypothesis that SCI patients with a high level of sensorimotor impairment would show affected processing of their own emotions due to the decoupling between the periphery and the CNS. To test this hypothesis, they assessed acute SCI patients within one year from the lesion, with both a task evaluating facial expression in others and a task evaluating the ability to judge their own emotions in response to emotional stimuli of various valence (IAPS scenes). They found the SCI patients to be no different from healthy controls in their ability to recognize the facial expressions of others. However, the authors found statistically significant differences in the ability to judge their own emotions when exposed to scenes showing anger and fear, notwithstanding that the intensity ratings of their own emotional response did not differ from the intensity ratings of the healthy controls. The current study differs from the one above, as we utilized a modified version of the previously used MET task and used normative data from the IAPS (means and standard deviations) to define the valence of a scene as positive, neutral, or negative. We then asked participants to evaluate the valence of images without specifying any emotional label. Furthermore, we asked participants to rate their direct emotional empathy (“how strong is your concern for the person in the scene?”) and their indirect emotional empathy (“how calm/aroused are you for the person in the scene?”). Specifically, the tasks used in the study by Pistoia et al. and the tasks used in the present study are fundamentally different, with the former forcing participant choices to set emotional labels and the latter looking exclusively at the ability to judge the valence of images as established by IAPS normative data.

Nevertheless, our results are in line with those of Pistoia et al. and, more generally, with peripheral theories of emotion according to which bodily information plays a pivotal role in the development of emotions as specifically addressed by the concept of embodiment [27, 37, 38]. These theoretical models introduced the concept of simulation as a fundamental component of emotional processing. According to the simulation theories, the reenactment of sensory and motor components of the emotional experience contributes to both the representation of the individuals' own emotional states and the ability to attribute emotions to others [39, 40]. Interestingly, bodily based simulative mechanisms interact with multiple cognitive-based processing modalities of emotional information [41]. Such interaction appears to be involved in the genesis of complex dissociations between the processing of the individuals' own emotional states and the ability to attribute emotions to others [16, 39, 42]. Within this interpretative framework, it is possible that SCI patients rely on a cognitive empathy strategy (hence higher perspective-taking) to adopt the perspective of others when they experience difficulties in evaluating an emotional condition. In other words, patients solve a basic task (valence attribution) in which they are impaired, by utilizing a cognitive, higher-order compensatory strategy. An alternative potential explanation could rely on the association of a general emotional blunting with depressive injury-related symptoms [33], as endorsed by the finding of a negative association between valence attribution accuracy and BDI scores. However, the BDI is not a clinical scale validated to formulate a clinical diagnosis of depression; it only investigates the presence of depressive symptomatology. None of the patients was indeed clinically assessed for depression, and this is a limitation of this study.

In the present study, we also showed that SCI patients gained higher scores on the *Perspective-Taking* scale of IRI as compared to the healthy controls and that their valence attribution accuracy was negatively correlated with self-reported ability to take the perspective of fictional characters (*Fantasy*) and, more generally, with the cognitive empathy component of the IRI. Together, these findings suggest that SCI patients tend to rely on a cognitive empathy strategy when trying to define the valence of complex emotional situations. That is to say, when analyzing the meaning of emotional stimuli, SCI patients tend not to rely on the emotion simulation pathway, but rather on a more cognitive-based strategy corresponding to cognitive empathy [3].

Although emotional empathy is not overtly impaired in SCI patients as a total group, it seemed to become progressively less efficient with increasing time from lesion, thus favouring the shift towards a cognitive-based strategy to analyze emotional stimuli like the IAPS complex scenes. It has been demonstrated that IAPS pictures elicit in the observer significant heart rate and skin conductance changes [43], as well as changes in the functional activity of brain structures playing a key role in the processing of emotional and visceral (interoceptive) information [44]. Since interoceptive signals are crucial for appraising emotional stimuli, reduced efficiency of interoceptive-mediated pathways in SCI would lead the patients to rely more on cognitive-based processes when interpreting emotional stimuli. However, here, we have been able to demonstrate that this cognitive approach in appraising emotional situations could reduce efficiency of valence identification when dealing with complex affective information.

This study has some limitations. First, we used static images taken from the IAPS and attempted to elicit emotional empathy responses. The IAPS is a collection of pictures with normative data for valence and arousal. It constitutes a great resource for experimental studies; however, the pictures were taken more than a decade ago and therefore are somewhat dated and sometimes hard to relate to. Furthermore, the use of static stimuli does not represent the ecological expression of emotions, which are dynamic by nature. Another potential confounder in the study is the variability in time elapsed since the lesion in the SCI group. We can indeed recognize two subgroups of patients in our sample: one with lesions that occurred within one year of testing and another chronic group with lesions that occurred long before the testing session (see patient characteristics in Table 2). Future studies should follow up longitudinally SCI patients to clarify how impairments in the processing of emotions evolve and adapt with the passage of time. Moreover, further studies on this issue should investigate the presence of any cognitive dysfunctions in patients through structured neuropsychological tests, as subtle cognitive deficits might not be captured through the traditional neurological assessment, which has been performed in our patients.

Overall, these results shed light on the ability of SCI patients to execute low-order and high-order emotional processes. The finding of a compensatory cognitive strategy that these patients may use when appraising complex emotional stimulus can be a promising start point for future studies investigating cognitive training to improve the emotional and social life of these patients. This is particularly relevant if we consider the increasing incidence of SCI, not only in young subjects but also in older people. The detection of impairments in emotional modulation is worthy of further investigation to plan tailored rehabilitation approaches and integrated strategies to enhance patients' compliance with care and rehabilitation.

## Figures and Tables

**Table 1:** Questionnaires' means and standard deviations and comparisons between SCI patients and controls.

	SCI patients (*n* = 20)		Controls (*n* = 20)		Comparisons
	Mean	SD	Mean	SD	*t* (*p*)
STAI-Y: State	40.8	15.6	34.1	8.4	1.698 (0.098)
STAI-Y: Trait	41.5	11.1	38.0	7.7	1.154 (0.256)
TAS	45.6	13.0	47.4	8.3	-.503 (0.618)
BDI	6.2	4.9	4.2	2.1	1.706 (0.096)
IRI	83.3	11.3	87.7	10.4	-.693 (0.492)
IRI *Fantasy*	17.8	5.4	19.7	6.1	-1.006 (0.321)
IRI *Perspective-Taking*	24.6	3.7	21.2	4.0	2.751 (0.009)
IRI *Personal Distress*	16.7	5.5	19.3	4.6	-1.580 (0.122)
IRI *Empathic Concern*	26.1	4.5	27.5	3.2	-1.113 (0.273)

*Notes:* SD = standard deviation; TAS = Toronto Alexithymia Scale; BDI = Beck Depression Inventory; IRI = Interpersonal Reactivity Index.

**Table 2:** SCI patients' characteristics.

*n*	Sex	Age	Type	Level	ASIA impairment	Time from lesion
1	M	67	Vascular	C2	A	9 months
2	F	65	Degenerative	C4-C6	C	48 months
3	F	59	Vascular	D5-D6	C	24 months
4	M	63	Traumatic	C4-C5	C	1 month
5	F	75	Vascular	D6	B	1 month
6	M	22	Traumatic	C5-C7	A	24 months
7	F	46	Traumatic	C4-C5	A	7 months
8	F	30	Traumatic	C6-C7	A	3 months
9	F	77	Traumatic	C4-C6	B	1 month
10	M	56	Traumatic	C5	B	504 months
11	M	57	Traumatic	C5	A	372 months
12	M	33	Traumatic	C5	B	192 months
13	M	53	Traumatic	C4	A	108 months
14	F	37	Traumatic	C4-C6	B	60 months
15	M	56	Traumatic	C3-C4	B	84 months
16	M	36	Traumatic	C5-C6	A	156 weeks
17	M	65	Traumatic	C5	B	240 weeks
18	M	44	Traumatic	C5	B	<1 year
19	M	52	Traumatic	C3-C6	B	3 months
20	F	26	Traumatic	C1-C7	B	4 months

## Data Availability

The data used to support the findings of this study are available from the corresponding author upon request.
